# Skin Grafting1Read before Buffalo Medical Union, November 28, 1900.

**Published:** 1901-06

**Authors:** F. B. Rasbach

**Affiliations:** Buffalo, N. Y.


### SKIN GRAFTING.[1]

By F. B. RASBACH. M. D., Buffalo, N. Y.

IN retrospecting the advancement in surgical science—both in application and technique—in the latter half of the nineteenth century, one must surely recall that of skin grafting, and those of us in Buffalo should be duly mindful of this simple, though extremely useful operation; for it was at the Sisters' Hospital, in this city, that the first successful attempt of skin transportation was made.

With your indulgence it may be profitable to here review the history of skin grafting, watching its various steps, and the changes it has passed through. History, and especially medical history tell us that during the Grecian and Roman wars medical men of these early times replaced by careful adjustment severed noses, fingers and ears successfully establishing the continuity of these injured members for their cosmetic effect, if not for their usefulness. Over a century ago John Hunter of England succeeded in transplanting a spur from a cock's leg into the comb of a second fowl and found that it not only lived but grew.

In 1847, Frank Hastings Hamilton, in his clinic at Geneva, N.Y., offered to a patient with an ulcer of the leg, which was exceedingly troublesome in healing, the advantages of an operation whereby he was to take the skin from the opposite leg and transplant it to the seat of the ulcer of the affected leg. This operation, however, was refused, but in 1854, seven years later, in the Sisters' hospital in this city, he put his ideas into practice by an operation on Horace Driscoll, by transporting a piece of skin of his left leg to an ulcer of his right leg, which he reported in December of that year in *Buffalo Medical Monthly Reporter*, now the BUFFALO MEDICAL JOURNAL.[2] This is the first successful attempt at skin grafting recorded. This operation was accomplished by dissecting a large piece of skin, 7x4 inches, from the selected site, taking all its layers down to the fascia, but leaving a nutritive pedicle or base. Between the skin and fascia he interposed a piece of lint, to prevent its uniting with the surface from which it had been lifted. Two weeks afterwards, when granu-

1. Read before Buffalo Medical Union, November 28, 1900.

2. The full title of this journal at that time was: *Buffalo Medical Journal and Monthly Review of Medical and Surgical Sciences.*—EDITOR.

lations had formed on the cut surface, he prepared the ulcer of the right leg for the reception of the flap by dissecting out the granulations and part of the cicatrix, forming a deep bed, into which he inserted the flap after removing the granulations from its under surface. The legs were then fastened together, and two weeks later, making four weeks in all, the pedicle or base was separated from the left leg the flap having united throughout most of its edge and under surface. By this operation he taught these principles, that the skin grew from the graft as well as from the margin of the ulcer and that the graft stimulated the margin to proliferate.

In 1869, Reverdin, an interné in Charity Hospital, Paris, reported in December of that year a case he had successfully operated upon by the following method, which is to use minute pieces of the outer layers of healthy skin; the pieces removed should only be the size of the head of an ordinary pin and only the epidermic layer should be taken; the cut surface should not bleed. The rete-Malpighii is the layer sought for application to the granulations. These grafts are placed about one-fourth of an inch from the margin on the ulcer, and at an equal distance from each other.

In 1880, Fisher, of Strassburg, devised a method somewhat resembling Hamilton's, only the grafts were entirely detached and both ulcer and site from which the skin sections were to be taken, were rendered bloodless (ischemic) immediately before the grafts were cut, by an Esmarch or an elastic bandage, and the grafts were applied immediately to the ulcer, which had been duly prepared. He found that ulcers treated by this method could be completely covered by pieces of skin of almost any size, and that the ulcer healed with little or no secretion; the dissolution and decomposition of the external layers of the grafts did not take place and the wound remained dry. This method is most feasible in hospitals where amputations are performed, the severed members being utilised to furnish the grafts.

Then followed Thiersch, in 1886, whose method is now so universally used because of its uniformly good results and its simplicity, and the fact that in most cases the patient himself can furnish the grafts and without producing a second ulcerating surface. In this method grafts are only cut through the papillary layer, and it is based on the viability of the superficial layers of skin when entirely removed from their original source of nutrition, and their tendency to form permanent attachments to a denuded surface. In the technique of the operation he follows Lister's principles of strict asepsis, both of the ulcer and of the site from which the grafts are taken, which are preferable from the thigh, leg, or arm, and whichever site is selected it is wise to shave, wash and leave in aseptic dressings at least twenty-four hours before the operation, preparing the ulcer at the same time, which should have healthy granulations. It has been my custom for some days previous to the operation to use as a wash for the ulcer a two per cent. zinc sulphate solution and a gauze dressing mounted in the same solution, which stimulates the ulcer and cuts down the exuberant granulations, making a good bed for the reception of the grafts, relieving the necessity of curretting, and subsequent hemorrhage at the time of the operation.

The grafts should be thin, long ribbons, taking just the epidermic layers, and the site from which they are taken should not bleed; use a razor, held nearly parallel with the surface to be cut, and during the cutting, which is done by a sawing motion, keep dripping on the razor a 2 per cent. salt solution, from gauze soaked therein, which assists the cutting by keeping the grafts from sticking and curling on the razor.

Dr. Weir, of New York, has devised some hooks to keep the skin taut, so as to cut larger, *i. e.*, wider and longer strips. I find by taking a thin piece of wood and some small tacks, upholster or gimp tacks, and driving them through the wood so that they just protrude, one can make a rake, easily sterilised, which answers all purposes. The grafts should be applied immediately to the ulcer, pressed gently but firmly down and when the ulcer is entirely covered, apply strips of gutta-percha protective, one over-lapping the other. Next, gauze soaked in salt solution and, outside of abundant gauze dressing, a sheet of gutta-percha protective, to keep the dressings moist, which should be moistened with the salt solution from time to time until the third day, when the first dressing is made. Care should be taken not to disturb the grafts, but if any secretion is present, gently loosen and float the gutta-percha tissue strips free, and wash thoroughly with the salt solution.

The grafts may take en masse, or at the end of a week or ten days look as if they were loosened and macerated and wash off easily. Do not rub them off in despair. Keep up the gentle washings and same precaution in dressing, and in a few days the field will be studded with little white spots; these are little islands composed of epithelium cells and they will proliferate quite rapidly, and though the granulations become exuberant between them, they spread out until they become attached to their neighbors, and in the end will choke or strangle these granulations in their meshes. The surgeon can again assist nature by clipping these high points of strangulating granulations, stopping hemorrhage by a point of silver nitrate, and in a short time the surface will be covered with a thin layer of skin. This needs protection for a time and by the application of some emollient it will thicken somewhat. However, it will never become as tough as natural derma; no hair will grow on it, and no sweat or sebaceous matter come from it, and little or no superficial fascia form beneath to cushion it, though its attachment will be firm. Therefore it is not applicable to places needing resistance to great pressure, as palms of hands or soles of feet. These varieties need to be grafted by Wolf's method, or if on the hand by the "hip pocket method," which I will not here describe.

Following Thiersch and adopting his technique to a greater or less extent, different operators began using the skin of animals, such as pig, dog, rabbit and frog, and though there are many reported successes, they are not as satisfactory as Thiersch's method, but have some utility when very large surfaces are to be covered.

At the meeting of the New York State Medical Society, in 1895, Dr. Lusk, of Warsaw, read a paper, detailing a method he had used in a case of extensive burn. Using some of the exfoliated epithelium, the result of vesication, that had been separated from its normal attachments for five weeks, he softened it in warm boric acid solutions, and used small pieces, *i. e.*, making twelve grafts from a piece an inch square, and seven of the twelve took. He believes that if these pieces are kept at a proper temperature, 40 to 90° F., they will keep almost indefinitely. He has since extended the practice by raising a blister with cantharides using the exfoliated epithelium, and he reports this method very successful, there being a marked absence of cicatricial contraction and scar tissue is firm and well nourished.

Amat, in 1895, reported using the lining membrane from shell of an egg for grafting extensive burns, and reports favorably of the method in this class of cases.

It has not been my privilege to try all these methods, in fact, I can only speak by experience of two—Reverdin and Thiersch—and I consider the latter the better.

Within the last three months, I had an opportunity of using conservative surgery on the hand of a young man, which had been crushed and badly lacerated between two rollers in a small printing press. The hand was properly denuded and dressed, and at the end of four weeks there was a large ulcer on the palmar surface, extending from the lower third on the outer side and from the upper third on the inside, down over the wrist. To allow this ulcer to heal by old methods would have caused so much contraction as to impair the usefulness of the hand, but by Thiersch's method the hand is flexible and supple, and leaves the patient able to resume his former position in the shop with no impaired motion.

Thiersch's method has been a balm to the sufferer, and solace to the surgeon, for those cases which in the past could be afforded no relief were an aggravation and an annoyance to the surgeon, can today, by this positive method be relieved and the patients allowed great freedom in following their occupation.

172 ALLEN STREET.

